# Unusual Case of Aortic Coarctation Complicated by Mycotic Pseudoaneurysm and Bicuspid Aortic Valve Endocarditis

**DOI:** 10.5812/cardiovascmed.13838

**Published:** 2014-02-24

**Authors:** Niloufar Samiei, Azin Alizadeh, Arash Hashemi, Yalda Mirmesdagh, Kambiz Mozaffari, Saeid Hosseini

**Affiliations:** 1 Heart Valve Disease Research Center, Rajaie Cardiovascular Medical and Research Center, Iran University of Medical Sciences, Tehran, IR Iran; 2 Echocardiography Research Center, Rajaie Cardiovascular Medical and Research Center, Iran University of Medical Sciences, Tehran, IR Iran; 3 Rajaie Cardiovascular Medical and Research Center, Iran University of Medical Sciences, Tehran, IR Iran

**Keywords:** Aortic Coarctation, Aneurysm, False, Endocarditis

## Abstract

Coarctation complicated with mycotic pseudo-aneurysm is very rare. We are reporting a case of a 26-year-old man suffered from this pathology. As the incidence of mycotic pseudo-aneurysm is very rare in patients with aortic coarctation, the choice of this pathology for a patient presenting with unexplained fever is the only way to reduce the mortality risk.

## 1. Introduction

Coarctation of the aorta (CoA) is a relatively common defect which accounts for 5% - 8% of all congenital heart defects. It may occur in association with various other lesions, most commonly bicuspid aortic valve (1). Bicuspid aortic valve endocarditis associated with coarctation can cause high incidence of mortality. In addition, coarctation could be complicated with mycotic pseudo-aneurysm which is very rare. Herein, we are reporting a case of a 26-year-old male with coarctation and bicuspid aortic valve complicated by mycotic pseudo-aneurysm and aortic valve endocarditis at the same time.

## 2. Case Presentation

A 26-year-old man was referred to our hospital from a different hospital with primary diagnosis of infective endocarditis. His chief complaints had started 6 months before the admission time with unexplained fever and approximate 15 kg weight loss. He had no history of cardiac disease and his condition did not improve even after several different medical consultations and treatments. In the end, the patient was referred to our center after the detection of a new murmur in the heart. Physical examination revealed cachectic appearance and low grade fever (38ºC). The blood pressure was 135/80 mm Hg and his pulse rate was 88 bpm. Given cardiac auscultation, he had 3/6 systolic decrescendo murmur best heard in left sternal border and diastolic blowing murmur at the same site. Pulses in the lower extremity were weak with delay in comparison with upper extremity pulses. The other aspects of physical examination were unremarkable.

**Figure 1.**
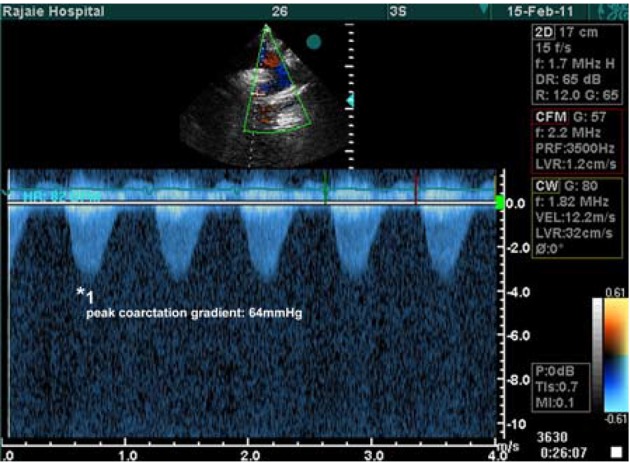
Continuous Wave Doppler in Coarctation Site

He underwent transesophageal echocardiography (TEE) showed gross left ventricular (LV) enlargement (LV volume = 273 mL) and mild to moderate systolic dysfunction and mild right ventricular (RV) enlargement with mild dysfunction. LV ejection fraction was reported about 45%. He had functional bicuspid aortic valve with right coronary cusp and left coronary cusp fusion at its base and severe aortic insufficiency (AI) due to lateral cusp perforation with moderate size vegetation (1*0.7 cm). Sub-aortic and non-obstructive web with peak gradient of 25 mmHg was also reported. In addition, TTE revealed coarctation of aorta (juxta ductal 64 mmHg) (Figure 1) with post stenotic dilation and multiple mobile vegetations (max size 1.5 cm). 

**Figure 2.**
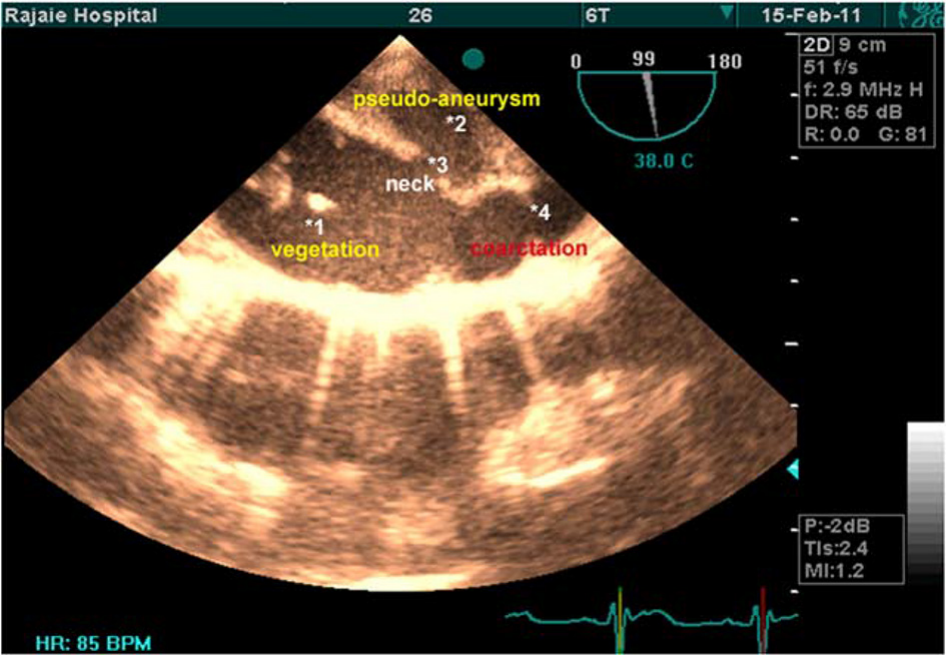
Transeshophageal 2D Image of Descending Thoracic Aorta in Long Axis (Rotation by 90º)

Furthermore, there was a large pseudo-aneurysm (3.5* 2 cm) with narrow neck and large clot formation inside the anterior site (Figure 2). There was also mild tricuspid regurgitation and normal pulmonary artery pressure.

The result of the blood culture came back positive with alpha staphylococcus haemolyticus. The decision was made to prescribe antibiotic therapy first and then staged surgical procedure. For twenty eight days, he took antibiotics (Vancomycin and Ampicillin/sulbactam) therapy. Afterwards, he underwent correction of coarctation and resection of the pseudo-aneurysm. As the our patient’s infection was well under control for one month by efficient antimicrobial therapy, the decision was made to use the prosthetic graft (Dacron tube) to replace the diseased part of descending aorta. Following femoral artery and vein cannulation, left posterolateral thoracotomy was performed. With the help of limited CPB (1 L/min), proximal and distal control of pseudoaneurysm, first a Dacron graft (20 mm) was interposed between distal part of aortic arch and thoracic aorta, approximately 2 cm distal to the pseudoaneurysm. Afterwards, the wall of pseudoaneurysm was opened and all the vegetation and inflamed tissues were drained as far as possible.

Finally, eight days later the patient underwent the final correction with aortic valve replacement for a bicuspid aortic valve. Following sternotomy and on cardiopulmonary bypass with moderate hypothermia, aortic cross clamp and introduction of blood cardioplegia, ascending aorta was opened. There was vegetation on aortic leaflet and due to aortic endocarditis, aortic leaflet was destructed. Therefore, all the aortic leaflets were excised and a St Jude prosthetic valve (regent, size: 23) was implanted. After the closure of ascending aorta and de-airing, aortic cross clamp was removed and in the end she was weaned off from CPB successfully (CPB time: 99 minutes, aortic cross clamp time: 62 minutes). After the surgery, antibiotic treatment continued for another 2 weeks. The result of the last TEE before his discharge did not show any abnormality at the level of the Dacron tube. Aortic valve prosthesis functioned normally without any vegetation, but there was persistence of severe LV enlargement and dysfunction (LVEF = 40% - 45%). There was also mild RV enlargement with moderate dysfunction. The patient was discharged from the hospital in good general condition.

## 3. Conclusions

The patients with aortic coarctation are at risk for endocarditis via the same pathogenic mechanisms by which valvular heart disease predisposes to the development of endocarditis. Severe aortic complications like mycotic pseudo-aneurysms are mostly occurred in adults with repaired or non-repaired coarctation of the aorta (1, 2). In a review of literature (1966 - 2004) by Anderson et al., 19 adult patients were identified with endarteritis of an aortic coarctation. The bacterial pathogens that cause coarctation endarteritis are the same pathogens which are known to cause valvular endocarditis including viridans group streptococci, coagulase-negative staphylococci, HACEK organisms and Staphylococcus aureus (3). Extra anatomic or in situ reconstructions are the two different surgical techniques to repair mycotic pseudo-aneurysm. The choice of procedure is usually surgeon-dependent. For in situ reconstruction, the use of aortic allograft is preferable to replace the infected tissues to decrease the incidence of late postoperative infection rate (4-7). In the case under study, the decision was made to use the prosthetic graft (Dacron tube) to replace diseased part of the descending aorta. As the incidence of mycotic pseudo-aneurysm is very rare in patients with aortic coarctation, the choice of this pathology for a patient presenting with unexplained fever is the only way to reduce the mortality risk.
